# Nuclear Envelope and Nuclear Pore Complexes in Neurodegenerative Diseases—New Perspectives for Therapeutic Interventions

**DOI:** 10.1007/s12035-020-02168-x

**Published:** 2020-10-17

**Authors:** Naomi Hachiya, Marta Sochocka, Anna Brzecka, Takuto Shimizu, Kazimierz Gąsiorowski, Katarzyna Szczechowiak, Jerzy Leszek

**Affiliations:** 1 Tokyo Metropolitan Industrial Technology Research Institute, Tokyo, Japan; 2 Laboratory of Virology, Department of Immunology of Infectious Diseases, Hirszfeld Institute of Immunology and Experimental Therapy, Polish Academy of Sciences, Wroclaw, Poland; 3 Department of Pulmonology and Lung Cancer, Wroclaw Medical University, Wroclaw, Poland; 4 Laboratory of Biochemistry, School of Veterinary Medicine, Azabu University, Sagamihara, Japan; 5 Department of Basic Medical Sciences, Wroclaw Medical University, Wroclaw, Poland; 6 Wroclaw Alzheimer’s Research Centre, Wroclaw, Poland; 7 Department of Psychiatry, Wroclaw Medical University, Wybrzeże L. Pasteura 10, 50-367 Wroclaw, Poland

**Keywords:** Nuclear membrane, Nuclear pore complex, Nuclear transport, Neurodegeneration

## Abstract

Transport of proteins, transcription factors, and other signaling molecules between the nucleus and cytoplasm is necessary for signal transduction. The study of these transport phenomena is particularly challenging in neurons because of their highly polarized structure. The bidirectional exchange of molecular cargoes across the nuclear envelope (NE) occurs through nuclear pore complexes (NPCs), which are aqueous channels embedded in the nuclear envelope. The NE and NPCs regulate nuclear transport but are also emerging as relevant regulators of chromatin organization and gene expression. The alterations in nuclear transport are regularly identified in affected neurons associated with human neurodegenerative diseases. This review presents insights into the roles played by nuclear transport defects in neurodegenerative disease, focusing primarily on NE proteins and NPCs. The subcellular mislocalization of proteins might be a very desirable means of therapeutic intervention in neurodegenerative disorders.

## The Role of the Nuclear Envelope, Nuclear Pore Complexes, and Nucleoporins in Nucleocytoplasmic Transport

### Nuclear Envelope

The nuclear envelope (NE) consists of two membranes: the outer and inner nuclear membranes (ONM and INM), which are bound by nuclear pore complexes (NPCs) and perforated by nuclear pores. Although the NE is a continuous membrane system, the protein composition differs significantly between the ONM and the INM [1]. The ONM is contiguous to the endoplasmic reticulum and sprinkled on the cytoplasmic side with ribosomes. At the same time, INM has indirect [2] interaction with a number of nuclear components, such as chromatin and nuclear lamina, which is an intermediate filament meshwork essential for the maintenance of the nuclear architecture [1, 3, 4]. The main function of NE is to protect the genome and ensure the safe transport of proteins between the cytoplasm and the nucleus [5]. Adequate functioning of the NE, in particular the INM, allows for the maintenance of the nuclear structure and position [6].

**Fig. 1:**
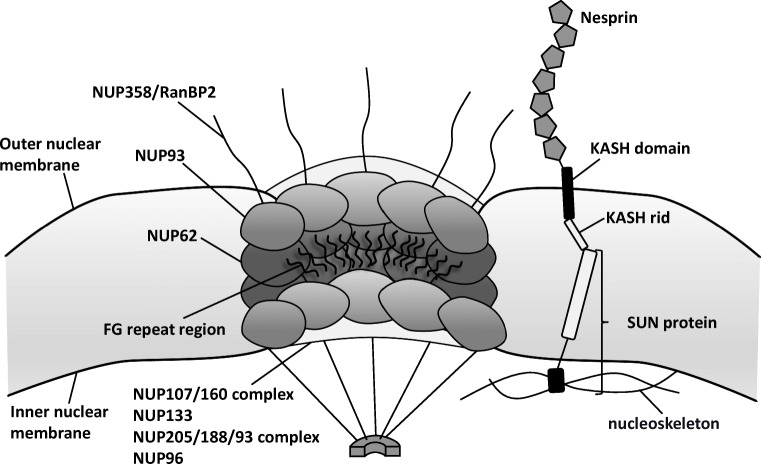
Schematic diagram of nuclear envelope (NE) and nuclear pore complex (NPC) proteins

Several NE transmembrane proteins, called nesprins, are characterized by the Klarsicht/ANC-1 /Syne-1 homology (KASH) domain (Fig. 1). These proteins are localized in the ONM and directly interact with the structural components of the cytoplasm and the Sad1-UNC-84 homology (SUN) proteins [7]. SUN proteins are integral membrane proteins and contain the linker of nucleoskeleton and cytoskeleton (LINC) complex, which tends to play a crucial role in nuclear positioning and movement and cell migration [8, 9] (Fig. 1). Well-coordinated nuclear movements appear to be essential during the formation of the central nervous system, where they function in both neurogenesis and neuronal migration. For example, as neural precursor cells migrate to the developing neocortex, the centrosomes travel continuously and ahead of the nucleus, accompanied by leaps rather than smooth, gradual transitions [10].

Nuclear lamina is an essential structural determinant of the entire nuclear envelope, connecting chromatin domains to the periphery of the nucleus and locating nuclear envelope proteins. The major components of the lamina are A-type and B-type lamins, which are members of the intermediate filament protein family. Expression of A-type laminate is controlled by development, while B-type laminate is present in all cells [11]. A-type laminate is formed by alternative splicing of the laminate A/C gene, and at least four protein types, A, AΔ10, C, and C2, are expressed. Lamin A is synthesized as prelamin, and mature lamin A/C is formed by the processing of 18 amino acids at the C-terminus. Lamin A/C interacts with chromatin and is involved in nuclear breakdown and remodeling during cell division and telomere dynamics. During somatic cell division, lamin A/C is phosphorylated and the structure of the network is degraded. As cell division comes to an end, it is dephosphorylated and reorganized into a network structure and the nuclear membrane is regenerated. Lamin A/C is known to interact not only with the nucleus but also with various proteins. It is also involved in the complex roles of the cytoskeleton and apoptosis, as well as in the replication, transcription, and transduction of DNA [12]. Alterations in A-type lamin often correspond to a selective disorder of peripheral nerves [13]. A recessively inherited missense mutation (R298C) in the LMNA gene encoding the A-type lamin rod domain causes axonal neuropathy that affects peripheral nerves, known as Charcot–Marie–Tooth type 2B1 disease. Sciatic nerves of LMNA-null mice display reduced axon density, increased axon enlargement, and the presence of unmyelinated axons; these characteristics are close to those seen in human patients and are consistent with the possibility that the R298C mutation may affect any aspect of A-type lamin function.

### Nuclear Pore Complexes

The majority of enzymes and their substrates that are engaged in cell proliferation, differentiation, and other vital functions are shuttled between intranuclear and cytosolic compartments through NPCs [14, 15]. The NPCs are aqueous channels formed by multicomponent protein complexes of the nuclear envelope, regulating the movement of cell components from the cytosol to the nucleus and vice versa. Consequently, defective NPC function could lead to inappropriate localization of a large number of nuclear and cellular components.

The NPC composition and structure are age-dependent, as cells lose essential nucleopore proteins with age. Cell cultures exposed to oxidative stress also show marked changes in phosphorylation and O-glycosylation of nucleoporins and alterations in the localization of these proteins and their interaction with other transport components. These types of changes in the structure and function of the NPC could lead to aberrant intracellular trafficking of crucial proteins involved in signaling and cell cycle regulation [4].

The NPCs show a broad degree of compositional and structural conservation in all eukaryotes. It has a doughnut-shaped structure consisting of eight spokes, which are arranged radially around a central channel that serves as the conduit for macromolecular transport [16–18]. Additionally, NPCs can interact with chromatin and contribute to the formation of specific genomic loops [19].

A growing body of evidence, both from human brain tissue and model animal studies, indicates that the disturbances of NPC structure and function resulting from neuronal oxidative stress are typical features of degenerating neurons. Thus, it can be hypothesized that abnormal structure and disturbed function of NPC underlie the pathogenesis of neurodegeneration.

### Nucleoporins

Nucleoporins (NUPs) are proteins that make up the components of NPCs [20–23]. Around 30 different NUPs are assembled together [24] (Fig. 1). The NUPs can usually be subdivided into two groups. The first category is the structural scaffold of the NPC, which is permanently embedded in the NE and contains the NUP107/160 complex and the NUP205/188/93 complex. NUP133, a component of the stable complex NUP107/160, is necessary for neuronal differentiation. Genetic deletion of NUP133 in mice causes lethality on approximately embryonic day 9.5 and a neural tube that does not normally close [25, 26]. The second class consists of “mobile” NUPs, including approximately 15 NUPs, which form peripheral components of the NPC and which shuttle off and on the NPC. Most of these NUPs have disordered phenylalanine–glycine (FG) repeat region [27], which binds directly to soluble transport receptors and facilitates transport via nuclear pore [28]. The disordered domains that distinguish FG repeats are characterized by a net positive charge, indicating that they represent a critical element of the selective barrier [2, 20].

In differentiated cells, such as muscle fibers and neurons, the oxidation of a long-lived NUP subset reduces the NPC turnover, which in turn increases nuclear leakage and may be responsible for age-related events [29, 30]. The NUP93 complex is selectively depleted from NPC in older cells. Its absence implies a potential mechanism for the degradation of the nuclear membrane barrier [31]. Nuclei purified from age-differentiated cells display defects in nuclear permeability and the accumulation of cytoplasmic tubulin, a finding consistent with the loss of nucleocytoplasmic compartmentalization.

The recessively inherited missense mutation of the NUP gene, NUP62, causes infantile bilateral striatal necrosis [32]. By comparison, the dominantly inherited missense mutation in NUP358/RanBP2 leads to an infection-induced, acute, necrotizing encephalopathy [33]. Although these are distinct diseases, they both include the acute development of bilateral necrotic lesions of deep brain structures. Interestingly, severe necrotizing encephalopathy is often caused by viral or other infections, and NUP96+/− mice have innate and adaptive immunity defects [34]. These intriguing reports indicate that the composition of the NPC can vary throughout development and in different tissues, and that particular proteins have tissue-specific functions, such as mediating nucleocytoplasmic transport of particular cargoes.

### Mechanism of mRNA Transport Through the Nuclear Pore

Nuclear export of mRNA is an essential process in eukaryotic gene expression. Immediately after initiation of transcription, mRNA binds to various proteins to form an mRNA–protein complex (messenger ribonucleoprotein: mRNP) [35, 36]. The mRNA precursor transcribed in the nucleus matures through complex processing such as capping, splicing, and 3′-terminal cleavage and poly(A) chain addition (cleavage and polyadenylation: CPA) [37]. Over 800 diverse protein groups function in the mRNA processing process such as transcription elongation, splicing, and CPA, and the binding of these many protein groups to mRNA is regulated in an orderly manner [35].

mRNA is recognized as cargo by nuclear export receptors by interacting with a group of RNA-binding proteins called adapter proteins of the mRNP components [37]. Premature mRNA precursors such as mRNA fragments during transcription and precursors that have not undergone splicing or CPA are eliminated from the nuclear export receptor cargo by coupling the recruitment cycle of the adapter protein with transcription and processing. Thus, the nuclear export receptor recognizes only mature mRNA as cargo, and the mRNA nuclear export mechanism plays an important role as one of the quality controls for mRNA [38].

Various RNAs expressed in cells are transported from the nucleus to the cytoplasm by their specific nuclear export receptors. Relatively small untranslated RNAs such as transfer RNA (tRNA), microRNA, and uridine-rich small nuclear RNA (UsnRNA) are transported from the nucleus to the cytoplasm by transport receptors belonging to the importin β family depending on Ran GTPase [39]. On the other hand, mRNA nuclear export depends on nuclear transport factor 2 (NTF2)–related export protein-1, Nxf1: Nxt1 heterodimer, a nuclear export receptor [40]. Nxf1 has RNA recognition motif (RRM), leucine-rich repeat (LRR), and NTF2-like domain (NTF2L) arranged in this order from the N-terminal. There is a ubiquitin-associated–like domain (UBAL) at the C-terminus. While NTF2L binds to Nxt1 to form a heterodimer, C-terminal UBAL shows the binding ability of NUP constituting NPC to the FG repeat sequence. Nxf1: Nxt1 heterodimer promotes mRNA passage through NPC by interacting with cargo and NPC via their respective domains [41].

### Protein Transport Between Cytosol and Nucleus

Two translocation signals are known to control the import of cargo proteins into the nucleus or vice versa: the nuclear localization signal (NLS) and the nuclear export signal (NES). Classical nuclear import requires the identification of NLS-bearing cargo by an import adapter. The importin α-cargo complex then travels through the NPC together with the importin β through NUP FG repeat interactions.

The small G protein, Ran, plays an important role in both import and export cycles. RanGTP binds to importin β on the nuclear side of the NPC in order to remove the cargo complex and release the cargo into the nucleus. Transport cycles include the replacement of the RanGTP nuclear pool, which is carried out by the import of RanGDP by NTF2 [42, 43]. The NTF2 binds to the NUP62 and is then actively transported to the nucleus via the NPCs [44]. Several NUPs in higher organisms are distinguished by the covalent addition of O-linked N-acetylglucosamine (O-GlcNAc) to serine and threonine residues [45]. O-GlcNAc glycosylation of NUPs is produced by oxidative stress, and this oxidant-dependent increase in O-GlcNAc modification can be accomplished by complex regulation of O-GlcNAc transferase and β-N-acetylglucosaminidase. This sugar content can mediate some of the interactions with the importins. For example, wheat germ agglutinine (WGA) is a lectin that binds to N-acetylglucosamine. When added to isolated rat liver nucleus, WGA can inhibit nuclear import by associating with sugar-modified NUPs, thereby blocking the channel [46, 47]. As a consequence, the modification of O-GlcNAc results in a nucleocytoplasmic import deficiency, especially in differentiated cells.

A 70-kDa heat shock protein (HSP70) is a cytoplasmic import factor for importin-independent nuclear imports [48]. The chaperone mechanism of Hsp70 acts to recover from protein denaturation in both the cytoplasmic and nuclear compartments of mammalian cells. Hikeshi, which interacts specifically with HSP70, can bind directly to NUPs, including FG repeats, and migrate into the nucleus via NPCs. Hikeshi-mediated nuclear import of Hsp70 is required to protect cells from heat shock damages so that the deficiency can lead to neurological disorders due to inadequate cell stress response [49].

### Liquid–Liquid Phase Separation in Nuclear Transport

The functional unit in the cell is an organelle surrounded by a lipid membrane. Membrane-less structures such as nucleoli that produce ribosomal RNA were thought to be rare; however recently, structures without membrane such as stress granules have been found. Furthermore, accumulated information revealed that proteins and nucleic acids are concentrated and compartmentalized in each membrane-less structure, and most of them are involved in neurodegenerative diseases [50].

The mechanism of protein and nucleic acid compartmentalization without the membrane partition is explained by liquid–liquid phase separation [51]. A sequence that easily causes phase separation is known as a low-complexity sequence (LC sequence) and is formed from a limited number of repeated amino acids. The LC sequence forms a naturally denatured region that does not have a fixed conformation and can be a structure without a membrane [52, 53]. Well-known proteins with LC sequences are the proteins that form the FET family (FUS, fused in sarcoma; EWS, Ewing sarcoma; TAF15, TATA-binding protein-associated factor 2N) [54]. Each of the FET family proteins has a similar domain structure, serine-, tyrosine-, glycine-, and glutamine-rich (SYGQ-rich) domain, called prion-like domain, and RGG repeat, which is a repeating sequence of arginine, glycine, and glycine. Also, it has an RNA-binding motif, a zinc finger, and a nuclear localization signal. The SYGQ-rich domain alone causes liquid–liquid phase separation [55]. Under physiological conditions, the LC sequence of FUS, which is the causative protein of amyotrophic lateral sclerosis (ALS), causes liquid–liquid phase separation due to the formation of a polymer that is unstable and easily dissociates in a concentration-dependent manner. On the other hand, if there is a disease-related mutation in this part, it changes from an unstable polymer to a stable polymer, causing aggregation formation [56].

FET family proteins accumulate in the cytosol when stress occurs in cells such as heat shock and DNA damage and becomes constituents of stress granules (SGs). SGs contain many proteins involved in neurodegenerative diseases. In addition, increased formation of SGs and breakdown of SG degradation mechanism are associated with neurodegenerative diseases such as ALS and frontotemporal dementia (FTD). A repeat sequence due to an abnormal extension of 6 bases (GGGGCC) in the untranslated region of the C9orf72 gene identified as the causative gene of ALS/FTD produces a cytotoxic dipeptide repeat (DPR). Recently, Zhang et al. revealed that the impaired nucleocytoplasmic transport in C9-ALS/FTD is due to the accumulation of nucleocytoplasmic transport factors such as Ran, importin, exportin, and NUPs into SGs [57]. Gasset-Rosa et al. found that endogenous levels of TDP-43 cause liquid–liquid phase separation in the nucleus. They also found that long-lived TDP-43 droplets formed in the cytoplasm were formed independently of conventional stress granules [58]. Furthermore, Kang and colleagues recently showed that tau, the cause of frontotemporal lobar degeneration (FTLD), condenses on the NE and inhibits nuclear–cytoplasmic transport. Interestingly, they also found that in living cells, tau on the nuclear envelope behaves like a droplet [59].

### Nucleoporins and Nuclear Envelopes in Chromatin Organization and Gene Expression

Although NPCs are known as the critical regulators of nucleocytoplasmic transport, more recent data suggest that these structures are essential for nuclear organization and that NUPs are extensively involved in genome organization and modulation of genome activity. Within the nucleus, chromosomes occupy distinct regions from which actively transcribing genes loop into structurally distinct interchromatin compartments. Genes at the nuclear periphery tend to be inactive, and alteration of their partitioning to the interior results in gene inactivation. Topological constraints required for looping are provided through the associations of discrete regions of the genome with the nuclear scaffold/matrix attachment regions (S/MARs), which provide anchorage for higher-order genome structure. The crucial factors for DNA processing, e.g., topoisomerases, are often found associated with the nuclear scaffold/matrix, and S/MARs function in augmenting transcription, facilitating replication and DNA repair, and insulating genic domains [60, 61].

Mounting evidence suggests that proteins of the INM, lamins and NE transmembrane proteins (NETs), play critical roles in chromatin tethering and regulation of gene expression [62, 63]. NE and NPCs can recruit chromatin with specific epigenetic marks of silenced gene expression; for instance, the hypoacetylation state of nucleosomal core histones is a well-known mark of silent chromatin, whereas acetylated histones mark active chromatin [64, 65]. NPC proteins also recruit gene-silencing factors or transcription-activating factors to chromatin sequestered at the periphery of the nucleus [66, 67]. NE and NPCs interact with chromatin with a preponderance of gene-silencing effects [34, 68], although several studies have reported NPC-proximal transcriptional activation with concomitant recruitment of induced genes to the nuclear periphery [34, 68, 69]. The role of NUP93 protein interaction with histone acetyltransferase (HAT) in gene silencing is well established [65, 68, 69], and activation of the interferon α and γ gene expression and regulation of interferon α- and γ-induced genes (MHC class I and class II) were documented for NUP96 [34, 68]. Thus, division of labor within different members of the NUP family, between silencing and activating gene expression, determines the complex role of the NPCs in gene regulation [65], and the NPC function should be perceived as a bridge between nuclear transport and gene regulation [70].

Neurons display a complex, highly polarized morphology; consequently, they could be particularly sensitive to age-related disruptions of cell nucleocytoplasmic trafficking and gene expression [71, 72]. Deterioration of the NPC composition in aging neurons could seriously change chromatin organization and function. For instance, NUP93 associated with the global histone acetylation profile is frequently damaged and lost during aging [16]. One could expect that significant changes in genome organization and gene expression, specifically those present in aged or degenerating neurons, will be revealed and quantified with modern molecular methodology.

In recent years, liquid–liquid phase separation of proteins has also been found on chromatin, which has a great impact on the research fields of chromosomes and chromatin. It is important that HP1 (heterochromatin protein 1) undergoes liquid–liquid phase separation for the formation of heterochromatin, a condensed and inactive chromosomal region [73]. In addition, it was revealed that the liquid–liquid phase separation involves phosphorylation of the naturally denatured region on the N-terminal side of HP1 [59]. In the transcriptionally active region, SAF-A (scaffold attachment factor A/hnRNP-U), an RNA-binding protein, forms oligomers through its ATPase activity and binding to RNA, and functions to preserve the decondensed chromatin structure. SAF-A has an RGG motif that is an LC sequence, causing liquid–liquid phase separation, but RNA binding prevents it [61].

## Nucleocytoplasmic Transport Impairment

### Polyglutamine Diseases

Polyglutamine (polyQ) diseases encompass a group of heritable neurodegenerative disorders, including Huntington disease (HD) and several spinocerebellar ataxias characterized by the pathogenic expansion of existing CAG trinucleotide repeats in the coding region of disease genes, which are translated into expanded polyQ domains in disease proteins [74, 75]. HD is a form of a polyQ repeat disease that leads to the formation of aggregates of polyQ-expanded huntingtin (Htt) protein in cell nuclei [76]. In the pathogenesis of HD, the mislocalization and aggregation of NUPs, as well as impaired nucleocytoplasmic transport, play an essential role [77]. Gasset-Rosa et al. found that age-related cellular characteristics, such as decreased nuclear envelope integrity, impaired nuclear–cytoplasmic transport, and accumulation of DNA double-strand breaks, were found in the cerebral cortex and striatum of HD model mice, depending on the amount of mutant protein and aging [78]. They also found that the accumulation of huntingtin-bound polyglutamine causes the structure of the nuclear envelope to collapse. At this time, Gle1, which is a major component of mRNA export necessary for nuclear–cytoplasmic transport [79], and RanGAP1, which is a Ran GTPase–activating protein [80], were partially segregated and mRNA accumulation occurred in the nucleus. In addition, major changes in nuclear morphology, irregular position of RanGAP1, and nuclear accumulation of mRNA have been observed in the cerebral cortex of HD patients as well as in the mice model. This indicates that polyglutamine-dependent inhibition of nuclear transport and altered nuclear integrity are central components of HD. Nuclear accumulation of Htt is associated with increased phosphorylation of Ser13 and Ser16 [81, 82] of the N-terminal of Htt and decreased interaction with the nuclear pore protein Tpr, which is involved in the nuclear export of proteins [7, 81, 83]. Huntingtin-lowering strategies may become promising therapeutic options in the future [84].

### Multiple Sclerosis

Axonal swellings, impaired axonal transport, and inflammatory demyelination are the hallmarks of multiple sclerosis (MS). The morphological changes are characterized by a succession of enlargements and constrictions along the axon and the detection of “ovoids” or “endbulbs” that resemble the terminal stumps of axons [85, 86]. Axonal damage has been associated with mitochondrial malfunction as well as related to calcium entry due to aberrant activation of sodium channels or activation by excitatory amino acids and cytokine production [87, 88]. Calcium-mediated nuclear export of histone deacetylase 1 (HDAC1) is a critical modulator of impaired mitochondrial transport [85] and the induction of axonal damage in inflammatory demyelination. Also, HDAC1 nuclear export is induced by pathological stimuli before impaired mitochondrial transport and the onset of morphological changes [89].

### Triple A Syndrome

AAA (triple A) is an autosomal recessive neuroendocrine disease in which esophagus achalasia, anhidrosis, and adrenocortical insufficiency are associated with muscular atrophy and weakness [90]. Adult triple A syndrome can have progressive neurodegeneration, Parkinson’s syndrome, and cognitive impairment [91]. ALADIN has been identified as the causative gene for triple A syndrome. It has been shown to be involved in many of the known aspects of the NPC function, including the assembly of NPC subdomains and the mediation of transport complex nucleation. No morphological abnormalities in the nucleus, nuclear membrane, and NPCs were observed in patients with triple A syndrome, suggesting that NPC dysfunction causes the disease. ALADIN-deficient NPCs are impaired in importin-dependent nuclear imports that prevent DNA ligase I and aprataxin, the protein required to repair DNA single-strand breakage. As a consequence, cells become susceptible to oxidative stress, resulting in cell death [92].

### Ataxia Telangiectasia

Ataxia telangiectasia (AT) is a hereditary disease with a major symptom of neurodegeneration and immunodeficiency. The causative gene is ataxia telangiectasia mutated (ATM), which is a member of the PI3-kinase family of serine/threonine protein kinase. The ATM protein is predominantly located in the nucleus at approximately 350 kDa. Histone deacetylase (HDAC) is an enzyme that eliminates acetyl groups from lysine residues present at the N-terminal of histones. Since the histone terminal from which the acetyl group has been removed binds to DNA, the chromatin structure becomes compact and the expression of the gene is suppressed. HDACs therefore play a central role in epigenetic transcriptional repression. To date, 18 forms of HDACs have been described in mammals and are categorized into groups I to IV on the basis of their structural differences. HDAC4, which belongs to class II, is highly expressed in the brain, especially in Purkinje cells. Typically, HDAC4 is located in the cytosol of neuronal cells since it binds to 14-3-3 proteins. On the other hand, brain tissues of patients with AT and Atm−/− mice have been shown to be localized in the nucleus. HDAC4 in the nucleus binds to myocyte enhancer factor-2 and cyclic AMP response element-binding protein (CREB) to cause histone deacetylase of chromatin, thus altering gene expression in neurons. In experiments using ATM-deficient mice, HDAC4 nuclear accumulation inhibition suppressed neurodegeneration and improved behavioral abnormalities [93].

### Amyotrophic Lateral Sclerosis

ALS is an intractable neurological disorder. The upper motor neurons of the cerebral cortex and the lower motor neurons of the brain stem and spinal cord are gradually degenerated and destroyed. Approximately 90% of patients are sporadic, with an unknown cause. Copper- and zinc-dependent superoxide dismutase (SOD1) gene mutations are responsible for the disease in patients with familial onset [94]. In experiments using mutant SOD1 (G93A) transgenic mice, the localization of nuclear–cytoplasmic transport proteins, such as importin α and β, changed their localization from the nucleus to the cytoplasm in the lumbar spinal cord. In addition, spinal cord anterior horn cells (AHC) have demonstrated a further increase in cytoplasmic localization of these proteins as the disease progresses [95, 96].

TDP-43 (TAR DNA-binding protein of 43 kDa), an RNA-binding protein, undergoes and accumulates modifications such as ubiquitination, abnormal phosphorylation, and fragmentation in the cytoplasmic inclusion bodies of denatured neurons. Approximately 90% of TDP-43 is initially found in the nucleus, but almost all of it is accumulated in cytoplasmic inclusion bodies in degenerated neurons. On this basis, research on the pathophysiology of the ALS cell death mechanism has been established on both sides due to the loss of function of the TDP-43 in the nucleus or the gained toxicity of the TDP-43 aggregate itself. FUS protein is an RNA-binding protein such as TDP-43 [97, 98].

Mutations in the FUS gene have also been identified in FTLD, indicating that RNA-binding proteins could be generally involved in the pathophysiology of ALS and FTLD [99, 100]. Many FUSs are located in the nucleus and participate in various RNA functions, such as splicing and stabilizing mRNA [101, 102]. It is known that the gene mutation of FUS identified in ALS is abundant near the prion-like domain on the N-terminal side and the nuclear localization signal on the C-terminal side [103]. Mutations that occur on the side of the nuclear localization signal can also change the localization of FUS from the nucleus to the cytoplasm, and patients with these mutations tend to develop earlier. FUS has a prion-like domain like TDP-43, so aggregates are easily formed, hence FUS-positive inclusion bodies are found in ALS with mutations in the FUS gene [99, 100].

As mentioned above (liquid–liquid phase separation in nuclear transport), the familial FTD-ALS linkage analysis reported a number of associations with the 21st region of the short arm of chromosome 9, and C9ORF72 was identified as a causative gene [104].

An abnormal extension of the hexanucleotide repeat sequence (GGGGCC) of the intron between exons 1a and 1b of C9ORF72 was discovered, and the number of GGGGCC repeats in healthy subjects is 2–23, while the number of patients has increased to more than 700 [69]. In this case, the symptoms are FTD, ALS, or a combination of both. As a clinical disease type of FTD, disinhibition type bvFTD is the most common. The primary pathology is a combination of FTLD-TDP and ALS, where positive TDP-43 inclusions are widely distributed in the cerebral cortex, hippocampus, basal ganglia, substantia nigra, brainstem, and spinal cord motor neurons. In addition to TDP-43-positive neuronal cytoplasmic inclusions (NCIs), TDP-43-negative and p62-positive NCIs also appear in the pyramidal cells of the hippocampus and cerebellar granule cells.

### Parkinson’s Disease

Parkinson’s disease (PD) is a progressive, age-related neurodegenerative motor system disorder that arises due to the loss of dopaminergic neurons in the *substantia nigra pars compacta* (SNc). Several examples point to an improper localization of various transcription factors and signaling molecules in dopaminergic neurons of diseased patients. Postmortem PD brains exhibit an increased nuclear translocation of nuclear factor-кB in the *substantia nigra* and ventral tegmental area neurons [105]. Activating transcription factor 2 (ATF2) is significantly downregulated in SNc neurons of PD brains [106], possibly indicating a decreased nuclear/cytoplasmic ratio of ATF2.

CREB is a family of transcription factors that formed the dimerized leucine zipper and is a transcription factor involved in memory, plasticity, and survival [103]. CREB is one of the most widely studied transcription factors in neurons, as more than 100 genes important for neuronal function contain the cAMP response element (CRE) in the promoter region [107]. In PD brain SNc neurons, pCREB aggregates are detected in the cytoplasm, while they are located in the control brain nucleus [108]. Since the nuclear activity of CREB plays a central role in neuronal adaptation and survival, segregation of this transcription factor in the cytoplasm may be a mechanism that contributes to neuronal degeneration and death.

TDP-43, which is usually a nuclear protein, was reported to accumulate in cytoplasmic inclusions in cases of FTLD and ALS but was also found in SNc neurons of PD patients, where it co-existed with Lewy bodies [109]. Overexpression of TDP-43 in the *substantia nigra* induced dopaminergic neuron death and potentiated α-synuclein toxicity to dopaminergic neurons in the PD mouse model [109]. The data suggest that cleavage of TDP-43 by caspases leads to its toxic gain of function; the truncated protein redistributes from the nucleus to the cytoplasm, where it joins with Lewy bodies [110].

### Alzheimer’s Disease

Alzheimer’s disease (AD) is the most common type of dementia which causes memory, thinking, and behavioral problems. Improper subcellular locations of various transcription factors and signaling proteins and karyopherins have been identified in the neurons of the AD brain. In AD brain neurons, the interface between the cytoplasm and the nucleus is significantly altered. Evidence of abnormalities in the nuclear membrane and aggregation of nuclear pores, intranuclear position of tubulin-positive filaments, accumulation of Nrf2 in the cytoplasm, reduced nuclear content of Nrf2 in hippocampal neurons, and decreased pCREB levels in AD brains [45, 71, 111, 112] strongly suggest impaired nucleocytoplasmic trafficking in affected AD neurons. Importin alpha was found in the Hirano body of AD neurons in human hippocampal CA1 neurons, but not in amyloid β plaques or neurofibril entanglement [98]. In addition, it was not observed in Lewy’s bodies in PD or in Pick’s bodies in Pick’s disease, indicating that this occurrence is unique to AD [112, 113].

Incorrect localization of TDP-43 in AD has also been found in the cytoplasm of inferior olive neurons where intracellular inclusion bodies are formed [114]. TDP-43 mislocation was detected in 25–50% of AD cases, particularly those with more serious clinical pathology [115]. It is worth noting that the existence of TDP-43 was also reported in healthy elderly people at increased risk of AD [115]. Postmortem brain study of nine patients with early mild AD showed elevated cytoplasmic ATF2 levels. Detection of the incorrect position of this primarily nuclear protein may be useful in the distinction between healthy and slightly impaired neurons [116].

GAPDH has emerged as an enzyme involved in various cellular processes [117, 118]. It not only functions in cytoplasm glycolysis but also plays an important role in other cell compartments, including the nucleus [117, 119, 120]. Oxidative stress causes GAPDH to undergo S-nitrosylation. In AD, GAPDH expression and nitrosylation are increased, presumably leading to elevated levels of GAPDH in the nucleus, which in turn promotes apoptosis [118]. As a result, oxidant-induced changes in GAPDH enzyme activity and intracellular distribution reduce energy supply and advance apoptosis in the brain of AD patients. Since GAPDH is an existing target for oxidative damage in many neurodegenerative diseases, oxidant-dependent changes in nuclear transport and the resulting increase in cell death may be common to multiple types of neurodegeneration.

Pathological protein tau, which is aggregated in AD patients, can directly affect NUPs, leading to disturbance of their structure, mislocalization, and impaired function [121]. Pathological tau-induced neurotoxicity is associated with impaired nuclear–cytoplasmic transport due to tau interaction with NPCs [122].

## Perspectives of Enhancing Nucleocytoplasmic Transport in Neurodegeneration

Impaired nucleocytoplasmic transport is widespread in neurodegenerative disorders, and the possibility of therapeutic approaches for improving transport abnormalities is widely discussed. Since nucleocytoplasmic transport control is complex, however, more basic research is still required to thoroughly elucidate the mechanism of action of drug candidates targeting nucleocytoplasmic transport disorders currently being studied [23].

The most studied intracellular and preclinical studies are groups of small molecules, called selective nuclear export inhibitors (SINEs), that block nuclear export. This group of compounds has been shown to form covalent bonds on the exportin1 (XPO1) with cysteine (Cys-528) inhibiting exports of nuclear protein and ribonucleic acid [123]. SINE induces temporary degradation of the XPO1 protein, which is degraded reversibly when the SINE compound stops. Cytoplasmic protein aggregates resulting from inhibition of nuclear exports are lysed and returned to the nucleus via the karyopherin alpha, karyopherin beta 1 and beta 2, and transporin 1 nuclear import receptors [124]. Of the compounds in this category, KPT-276, KPT-350, and KPT-335 showed important neuroprotective effects of primary cultured neurons and induced pluripotent stem cells (iPS cells) in vitro [77, 125]. More recent studies, on the other hand, have shown that transport of TDP-43 and FUS proteins from the nucleus to the cytoplasm is primarily passive diffusion and XPO1 is not involved [126, 127]. Consequently, at the present stage of knowledge, it is difficult to explain the role of SINE in the ALS/FTD.

As another therapeutic strategy, an importin role reinforcement approach is envisaged.

There are, for example, methods of protein engineering to produce compounds that enhance the affinity of β2 karyopherin to FUS proteins and get functionally enhanced imports [124, 128].

By improving the function of these nuclear import receptors, the lysis of aggregates such as TDP-43 protein aggregates and FUS containing proline–tyrosine nuclear localization signals can be improved.

Optimizing and modifying nuclear export/import proteins that are impaired in many neurodegenerative diseases may offer unique therapeutic options for treating fatal diseases in these incurable diseases.

## Final Remarks

The role of impaired nucleocytoplasmic transport in the pathogenesis of neurodegenerative disorders is essential [108]. The NE and NPC are responsible for the bidirectional trafficking of proteins, including many transcription factors, between the cytoplasm and the nucleus, as well as for the spatial organization of chromatin and the regulation of gene expression. Translocation of signaling proteins is crucially involved in the pathophysiology of neurodegenerative disorders. In postmitotic cells, such as neurons, several nuclear pore proteins, such as the NUP107/160 complex, are long-lived, and during their lifespan, they accumulate oxidative modifications [30, 129, 130]. As a consequence, progressive deterioration of the NPC structure and function is observed in aged neurons, with cytosolic proteins being leaked into the nucleus [129]. Impaired functions of the NE and NPCs are regular features of age-related neurodegeneration [30]. Recent attention has been drawn to the mislocalization process in neurodegenerative disorders like AD, PD, HD, and prion diseases. Considerable evidence has been accumulated to indicate that the earlier stages of the protein mislocalization process are more directly tied to pathogenesis than the filamentous protein aggregates. For example, the mislocalization of tau to dendritic spines has recently been reported to mediate a synaptic dysfunction that is associated with impaired brain function at the preclinical disease stages that immediately precede neurodegeneration [131]. P-tau interacts with Nup98, the displacement of this nucleoporin from the nuclear membrane into the cytosol, where it often locates in neurofibrillary tangles, while Nup98 in the cytosol enhances P-tau oligomerization and aggregation [121]. Thus, tau-induced mislocation of NPCs contributes to tau-induced neurotoxicity in AD [121]. It has also been hypothesized that an altered localization of transcription factors such as NF-kB, activating transcription factors 2, CREB, p53, E2F transcription factor, and NF-E2-related factor 2 might contribute to cell death commitment in several neurodegenerative diseases [71]. Mislocalized proteins frequently join cytosolic or intranuclear inclusion bodies, which are present in the majority of degenerating neurons. A better understanding of the mechanisms of nucleocytoplasmic transport and the establishment of the NE and NPCs’ contributions in chromatin organization and gene expression should lead to new approaches for therapeutic intervention in many diseases connected with NE and NPC malfunction, as well as in neuronal aging and neurodegeneration. Especially, from this point of view, modifying the disease-related subcellular mislocalization of proteins might be an attractive means of therapeutic intervention. In particular, cellular processes that link protein folding, cell signaling, and nuclear import and export to the subcellular localization of proteins have been proposed as targets for therapeutic intervention. For example, blocking translocation of signaling proteins in subcellular compartments such as NF-kB can be of exciting action for suppressing disease, e.g., AD in the very early stages of disease processes before clinical manifestation.

## Abbreviations

AD: Alzheimer’s disease
ALS: Amyotrophic lateral sclerosis
ATF2: Activating transcription factor 2
CREB: cAMP response element-binding protein
FTLD: Frontotemporal lobar degeneration
HAT: Histone acetyltransferase
HD: Huntington disease
HDAC: Histone deacetylase
Hsp70s: Heat shock proteins of 70-kDa
Htt: Huntingtin protein
KASH: Klarsicht/ANC-1/Syne-1 homology
LINC: Linker of nucleoskeleton and cytoskeleton
MS: Multiple sclerosis
NE: Nuclear envelope
NES: Nuclear export signal
NETs: Nuclear envelope transmembrane proteins
NLS: Nuclear localization signal
NM: Inner nuclear membrane
NPCs: Nuclear pore complexes
NTF2: Nuclear transport factor 2
NUPs: Nucleoporins
O-GlcNAc: O-linked N-acetylglucosamine
ONM: Outer nuclear membrane
pCREB: Phosphorylated form of CREB
PD: Parkinson’s disease
S/MARs: Nuclear scaffold/matrix attachment regions
SNc: Substantia nigra pars compacta
SUN: Sad1-UNC-84 homology
TDP-43: Transactivation response DNA-binding protein 43
WGA: Wheat germ agglutinin
